# The Effects of Physical Activity on Academic Performance in School-Aged Children: A Systematic Review

**DOI:** 10.3390/children10061019

**Published:** 2023-06-05

**Authors:** Joseph James, Andy Pringle, Stuart Mourton, Clare M. P. Roscoe

**Affiliations:** Department of Sport and Exercise Science, Clinical Exercise and Rehabilitation Research Centre, University of Derby, Kedleston Road, Derby DE22 1GB, UK

**Keywords:** physical activity, exercise, physical activity intervention, academic performance, academic achievement, cognitive functioning, children, school-aged children

## Abstract

Schools offer a unique environment to influence children’s physical activity (PA) levels positively. This study aims to systematically review the evidence surrounding how PA affects academic performance by analysing how the frequency, intensity, time, and type of PA mediate academic performance outcomes. This review was conducted using the PRISMA framework. Keyword searches were conducted in Science Direct, PubMed, and SPORTDiscus. Children that were obese, typically developing, typical weight, disabled, with a developmental disability, from a low socio-economic background, or an ethnic minority were included. A total of 19 studies were included, with a total of 6788 participants, a mean age of 9.3 years (50.2% boys, and 49.8% girls). Overall, 63.2% were nondisabled, while 36.8% were diagnosed with a disability. Two authors met, reviewed papers with regard to the inclusion criteria, and agreed on outputs to be included. Evidence suggests that associations between PA and academic performance were primarily positive or nonsignificant. PA levels of 90 min plus per week were associated with improved academic performance, as was PA performed at moderate to vigorous intensity. The optimal duration of PA was 30–60 min per session, whilst various sports induced positive academic effects. Importantly, findings support that PA does not have a deleterious effect on academic performance but can enhance it.

## 1. Introduction

It is widely acknowledged that regular physical activity (PA) is inextricably linked to a plethora of health benefits [1]. Extensive research advocates PA’s role in improving a person’s physiological wellbeing [2]. Conversely, numerous studies have documented the ill effects physical inactivity can have on one’s physiological health [1,3]; most notably, Warburton et al. [1] recognised that physical inactivity is a modifiable risk factor for a diverse range of diseases, which include cardiovascular diseases, bone and joint diseases, and chronic diseases such as cancer (colon and breast), obesity, and hypertension. Regarding psychological health, PA has also been identified as an effective means of alleviating mild to moderate depression, improving mood, and reducing anxiety symptoms [1,4]. It is also well established that regular participation in PA facilitates a child’s physical, psychological, and social development [5]. Vaqnguero-Solis et al. [6] stated that childhood participation in PA positively affects body mass index (BMI), morphology, fundamental movement skill competence, self-esteem, and social behaviours. These findings are consistent with the literature [7,8,9,10,11].

Physical inactivity prevalence in children across the UK is concerning; in 2018/2019, less than half (47%) of children aged 5–18 years met the current PA guidelines of 60 min of moderate PA per day [12]. Accordingly, it can be inferred that strategies to improve PA levels in children across the UK are an irrefutable necessity, not only to improve children’s current health status, but also to decrease the likelihood of obesity and other inactivity-related conditions. Given the compulsory attendance of children at schools and the significant amount of time children spend there each day, schools offer a unique environment to positively influence PA levels among children so that recommended PA levels are achieved [13]. Nevertheless, opportunism to increase PA levels within schools is increasingly finite [11]. In fact, some schools are decreasing time spent on non-academic subjects such as physical education (PE) to allocate more time to academic subjects such as mathematics or English [14]. This decline in PA opportunities in schools is primarily influenced by ever-increasing academic pressures placed on schools and educators to achieve within an attainment-focused curriculum [14]. However, research indicates such declines in PA are detrimental to the child, their physiological and psychological health, and potentially, their academic performance.

A growing body of literature has investigated the effects of PA (school-based, class-based, and extracurricular PA) on academic performance, and the results are widely debated. Notably, several studies advocate PA to improve academic and/or cognitive performance [15,16,17,18,19]. De Greeff et al. [20] supported this positive effect of PA on executive functions (inhibition, working memory, cognitive flexibility, and planning) and academic performance, and stated that largest effects are seen with interventions that implement continuous regular PA over several weeks. This was similar to findings by Barbosa et al. [21] who found a medium positive effect of PA on academic achievement as opposed to acute PA which demonstrated no benefits. Further research [22] saw deviations from this as they reported acute PA interventions significantly improved processing speed, inhibition and attention, whereas chronic PA interventions significantly improved processing speed, attention, cognitive flexibility, working memory, and language skills. However, studies conducted by Esteban-Cornejo et al. [23] found that the association between PA and academic performance was negative and very weak. Similarly, Daley and Ryan [24] reported no correlations between increased PA and academic performance; these findings are consistent with the literature [25,26,27]. Contrasting results across the literature suggest that future research within this area is necessary to bring clarity to the field. If it was evidenced that PA could be implemented in schools without being a detriment to academic performance; policymakers and schoolteachers may be inclined to implement a policy of regular PA within the school context. This would ensure children meet their recommended PA guidelines (60 min or more per day) for most of the week.

Several authors have recognised the uncertainty surrounding PA’s effect on academic performance. Rasberry et al. [28] explored the association between four school-based PA contexts (PE, recess, classroom PA breaks, and extracurricular PA) and academic performance across 50 peer-reviewed articles. The review concluded that positive or no associations between the interventions and academic performance were reported across the studies that assessed PE, recess, and classroom-based PA. Whereas extracurricular PA interventions differentiated from the aforementioned contexts, mainly reporting positive or no associations but some negative associations (2%). Overall, this review strengthens the idea that PA within schools is vital, with PA enhancing academic performance rather than detracting from it. Likewise, a study conducted by Singh et al. [29] investigated the relationship between PA interventions and academic and cognitive performance and reported inconclusive results. Notably, of the 11 high-quality studies included in the synthesis, 60% reported a significant beneficial effect of PA on academic performance, and 48% reported a significant beneficial effect on cognitive performance. Sember et al. [30] also reported mixed results between PA and academic performance; specifically, of the 44 articles included in the synthesis, 20 reported significant, positive effects, and 24 reported adverse or null effects on academic performance. More recently Peiris et al. [31] reported physical activity had mixed effectiveness on academic performance, e.g., a positive effect on spelling performance (*p* < 0.05) and foreign language learning (*p* < 0.01) but no significant effect on mathematics and reading, and no effect on cognitive outcomes. The mixed results reported across the aforementioned reviews reiterate the necessity for further research to contextualise the causes of such diversity in findings, and further extend the understanding of the mediators for associated outcomes, whether positive, negative, or insignificant. Barisic et al. [32] stated PA is a multifaceted behaviour encompassing frequency, intensity, time, and type (FITT). These domains individually affect physiological processes differently. Therefore, it is crucial to explore the domains of FITT, individually and combined, to understand the underlying mechanisms behind the associations [32]. Consequently, it is suggested that FITT should be incorporated in future reviews to elucidate the cause of associated effects and improve the applicability of results if utilised to inform future interventions.

The applicability of the reviews is also problematic. Rasberry et al. [28], Singh et al. [29], and Sember et al. [30] only included children or adolescents that were nondisabled, and Marques et al. [14] did not specify the characteristics of the included population. This has significant implications for the applicability of the results, as subsequent findings will be unrepresentative of the school community. Notably, schools are heterogeneous environments characterised by a diverse range of individuals, subcommunities, and cultures [33]. To ensure results are applicable and relevant to those wishing to utilise them (school communities and policymakers), authors should not exclude participants on the basis of specific characteristics (disabled, nondisabled, culture, background, race, religion, sex, gender reassignment, and socioeconomic status). Instead, it is suggested that future research should only exclude participants on the basis of age (too old/young), as this is genuinely representative of what occurs in the schools’ heterogeneous communities.

Although several published reviews explore PA’s effect on academic performance, the above-indicated inadequacies in study designs suggest that further research is necessary to bring clarity to the field [28,29,30,32]. Therefore, the primary aim of this current study is to conduct a broad examination of the literature surrounding PA’s effect on academic performance. However, unlike other systematic reviews of this nature, this study aims to extend the understanding of the causal factors related to associated effects, recognising a heterogeneous school community. This is achieved through documenting the FITT of PA intervention designs. Findings are used to disseminate an optimal frequency, intensity, type, and time of PA for improved academic performance.

## 2. Materials and Methods

### 2.1. Protocol

This review was carried out according to the guidelines of the Preferred Reporting Items for Systematic Reviews and Meta-Analyses (PRISMA) framework; we did attempt to register with PROPSERO, but they did not accept it as it was looking at the effect of physical activity on academic performance, and they did not support research looking at academic performance. We felt that the review has great importance and implications; hence, although not approved by the PRISMA process, we performed the review following the PRISMA guidelines to see if there is any effect of PA on academic performance.

### 2.2. Eligibility Criteria

The literature synthesis was carried out according to the Preferred Reporting Items for Systematic Reviews and Meta-Analyses (PRISMA) framework. The criteria for selecting studies for inclusion in this review were as follows: published in the English language (although origin was preferably diverse to draw generalisable results); peer-reviewed, longitudinal, experimental studies published between March 2012 and June 2022; studies that focus on school-aged children aged 6–14 years of age; incorporated a PA intervention as the dependant variable; examined outcome variables related to academic performance using one or more outcome measure (e.g., performance on standardised tests, on-task behaviours, and performance on executive functioning tests). Diversity between study cohorts was also a prerequisite for the literature synthesis; studies were screened and chosen on the basis of their applicability to provide diverse findings that are relevant to and representative of the heterogeneous school community; groups to be included were children that were obese, typically developing, typical weight, with physical disabilities, with a developmental disability, from a low socioeconomic background, or an ethnic minority. Moreover, only studies that detailed the frequency, volume, and type of PA of their intervention were included in this review. However, studies that also included the intensity of PA were most desired. These predetermined measures were essential for two reasons. Firstly, clear and measurable dependant variable(s) ensure that documented interventions can be applied and repeated if later utilised to inform future practice. Secondly, through a clear understanding of intervention protocol and its outcomes, authors can reach an informed conclusion surrounding optimal intervention design.

Several terms relating to academic performance were included in this review under the encompassing term, academic performance (Table 1). It is recognised that a number of these terms are independent entities that are not inextricable to academic performance itself. Nevertheless, they are included in this review due to their well-established association/link with academic performance. Most notably, academic performance has been found to have a strong association with intelligence [34], cognitive functioning [35], and executive functioning [36]. It is recognised that only including these outcome variables would be insufficient to determine exactly how PA affects academic performance. However, their incorporation in the study design alongside standard outcome measures related to academic performance (mathematics score, reading score, standardised test scores, and academic achievement) allows for a more comprehensive analysis of PA’s effects on academic performance, whereby the direct and indirect effects of PA on academic performance are evaluated. Publications were excluded from this review if they were unable to meet the eligibility criteria discussed above or if they focused on the effects of PA alongside other independent variables (such as nutrition interventions or workshops). If the study did not explicitly state the participating cohort, the intervention, outcome measures, or results, it was excluded. Moreover, experimental studies that did not incorporate a control group were excluded from the study (the necessity for a control group was pre-established to gain valid and objective conclusions from the data/studies provided). Studies that focused on the immediate effects of antecedent PA on academic performance were deemed ineligible. Meta-analyses, case studies, systematic literature reviews, review articles, and observational studies were excluded from this article. Studies that did not include significance values to support findings were also excluded from this review.

### 2.3. Study Selection

**Figure 1:**
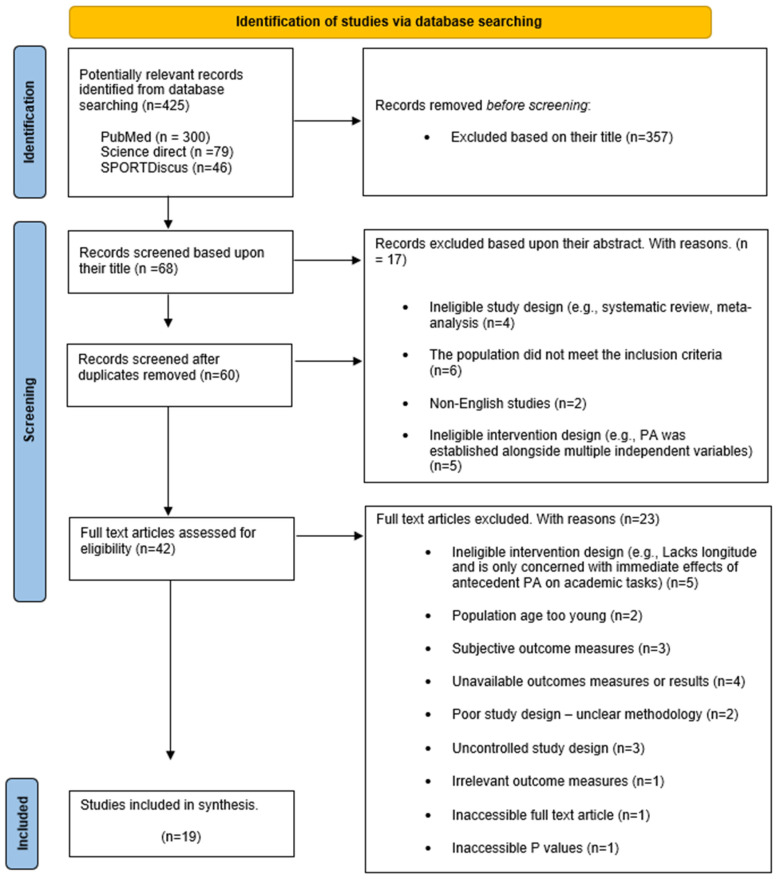
Visual depiction of academic performance (AP) and physical activity (PA) searches using the Preferred Reporting Items for Systematic Reviews and Meta-Analyses flow diagram.

The initial literature search using three electronic databases (Science Direct, PubMed and Sport Discus) yielded 425 results (the predetermined search terms used for this search can be found in Table 1). A total of 357 articles were immediately excluded on the basis of the title. Following this, duplicates were removed, and the abstracts of the remaining 60 studies were assessed for eligibility. Among the 60 articles, 18 articles were deemed ineligible. Accordingly, 42 articles remained for the full-text screening; 19 articles were considered eligible after the full-text screening and, therefore, included in the final synthesis. Conversely, 23 full-text articles were excluded for a variety of reasons; most commonly, articles were excluded because the intervention design did not adhere to the eligibility criteria (lacked longitude and mainly focused on the immediate effects of antecedent PA on academic tasks), outcome measures were either subjective (observational) or unavailable, methodology or intervention design was unclear and, thus, unsuitable for analysis, the study did not incorporate a control group/condition, or *p*-values were not provided to support relevant outcomes. See Figure 1 for a visual illustration of the study selection process discussed.

### 2.4. Identification of Studies

See Figure 1 for a depiction of the search strategy employed. Published studies were independently identified and assessed using three electronic databases (Science Direct, PubMed and Sport Discus) up to 9 March 2022. These databases were searched using pre-determined search terms related to PA, academic performance, and the target population (Table 1). Filters applied were concerning the year of publication (2012–2022) and study design (experimental studies, clinical trials, and randomised control trials). On completion of the initial search, the process of screening based on article titles was carried out. The titles were assessed on the basis of congruence with the eligibility criteria, and articles with ineligible titles were excluded from further screening. Duplicates were then removed, and the abstracts of the remaining articles were assessed for eligibility. When the article abstracts were screened, each article was evaluated and chosen for further assessment according to its ability to meet the eligibility criterion specified. However, if a study’s eligibility (based on reading of the abstract) was inconclusive, it was also included in the final screening. Lastly, full-text articles were screened for eligibility, and all articles that did not meet the inclusion criterion were removed. Subsequently, the remaining studies met the inclusion criteria and were included in the synthesis.

### 2.5. Data Extraction and Synthesis

An independent reviewer extracted the following data from the studies eligible for synthesis: author(s); study design; country of origin; sample (sample size, mean age of participants, and participant characteristics); the independent variable of interest (relating to PA) and intervention characteristics (FITT); dependent variable of interest and outcome measures (related to academic performance); relevant findings pertaining to academic performance and PA. Notably, there was no pre-established outcome measure sought. Instead, all outcome measures relating to academic performance-related keywords (Table 2) were documented. This holistic approach was adopted to ensure the different aspects of academia and in that academic performance was accounted for. The difference in curricula across the UK alone supports the necessity for this method, with differing pedagogies, subjects, and means of assessment from country to country [37,38,39]. It was evident that there was no encompassing measure to assess academic performance. Therefore, this article did not want to discredit or overlook any potentially significant/relevant outcome measures and their consequent findings due to stringent specificity that would not be exemplary of academia and academic performance itself. Due to the article’s narrative nature, any outcome or finding relevant to achieving this study’s objectives is documented and discussed. However, significance values are provided for each study to support statements regarding the significance of effects observed, if any. Moreover, no assumptions were made if a study did not include relevant data or findings in the text. Consequently, any study that did not provide relevant data or findings suitable for analysis was excluded from this article.

### 2.6. Analysis

A narrative analysis of the literature was adopted due to the diversity of populations, outcome measures, and multiple methods included. Moreover, at the time of the study, little literature surrounding PA’s effect on academic performance adopted a narrative approach; therefore, a narrative analysis was utilised to make a novel contribution to the field. A meta-analysis was not chosen due to the genuine differences between groups, interventions, and outcome measures that would otherwise not be considered if combined for meta-analysis. Instead, a narrative approach was most appropriate so that overall effects could be examined alongside providing opportunism to gain deeper insight into the intricacies and reasons for associated effects. Consequently, a comprehensive review could be achieved that, firstly, informs the reader of the overall effect PA has on academic performance and, secondly, objectively concludes the reasons for these effects.

## 3. Results

### 3.1. General Study Characteristics

**Figure 2:**
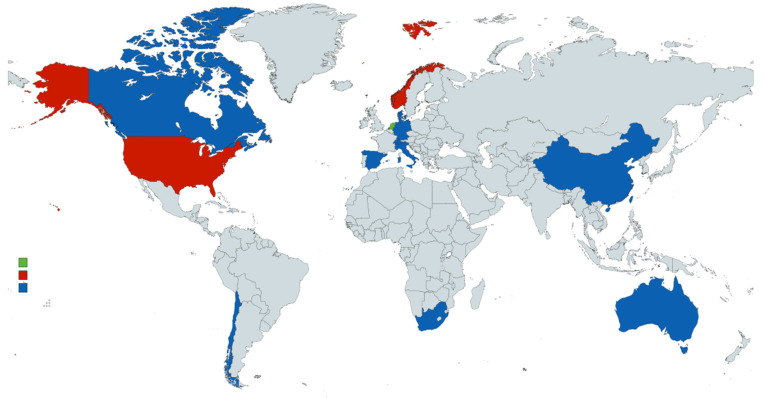
Visual representation of each article’s country of origin. Key: green shading—*n* = 3 articles; red shading—*n* = 2 articles; blue shading—*n* = 1 article.

A detailed description of the included studies, their characteristics, methodological processes, and key findings are summarised in Table S1. Of the 19 articles that met the eligibility criteria, the countries with the highest number of articles included were the Netherlands (*n* = 3), followed by the USA (*n* = 2) and Norway (*n* = 2). Then, the remaining articles represented one country of origin each, namely, Spain, Switzerland, Denmark, Chile, Australia, Taiwan, South Africa, Italy, China, Canada, and Germany. Figure 2 presents a visual representation of each study’s country of origin.

The total number of participants for the included studies combined was 6788. The average number of participants was 357, ranging from 22 to 1181. Of the 19 articles, 16 reported the mean age of the participants at baseline, which ranged from 8.01 to 11.35 years old, although the average age across the studies was 9.26 years. Moreover, among the 6788 participants that took part in the study combined, many were characteristically diverse. Most notably, 50.2% of the participants were boys, and 49.8% girls. Furthermore, 63.2% of the articles included participants that were nondisabled, whilst 36.8% included participants that were diagnosed with a disability. Specifically, the disabilities presented were autism spectrum disorder (ASD; *n* = 3), foetal alcohol syndrome (*n* = 1), and attention-deficit/hyperactivity disorder (ADHD; *n* = 3). Furthermore, a small number of the nondisabled studies presented stringent eligibility criteria related to participant characteristics; notably, Gall et al.’s [40] study only included participants from a low-socioeconomic background, whilst Reed et al. [41] only included participants from an ethnic minority.

Across the 19 interventions, PA was implemented through the following forms of PA; physically active academic lessons [40,41,42,43,44,45,46], PA lesson breaks [40,44,46,47], moderate intensity PA [48], moderate- to vigorous-intensity PA [42,43,45,47,49,50], high-intensity PA [48,51], cognitively engaging PA [47,49,51], aerobic exercise [51], mixed martial arts [52,53], table tennis [54], team games [51], PE lessons [40,41,44,46,48,49,51,55], PA homework [44], multi-activity sport [41,46,50,56,57], and PA that emphasises fundamental movement skill development [41]. Notably, several studies were categorised into various types of PA due to their multifactorial intervention design. For example, Ardoy et al. [48] implemented a multifaceted intervention design to compare cognitive and academic performance across three intervention conditions (group 1 received four 55 min periods of moderate-intensity PE per week; group 2 received four 55 min periods of high-intensity PE per week; control group received two 55 min periods of regular PE lessons per week); therefore, this study fell into various categories: moderate-intensity PA, high-intensity PA, and PE lessons.

### 3.2. Measurements of Academic and Cognitive Performance

Measurements of academic performance differentiated profusely across each of the included studies. However, this review categorised each study’s primary dependant variables into two encompassing domains: academic performance and cognitive performance. Notably, nine studies measured domains of academic performance, while 14 studies measured indicators of cognitive performance. Of the eight studies that analysed the effects on academic performance, four used grades in academic subjects [40,48,49,50]; meanwhile, three studies utilised standardised test results [43,49,55], one took measurements of the student’s mastery of the basic facts test [47], and one analysed mathematics and reading performance with the speed test arithmetic and the 1 min test [45]. Conversely, the majority of studies evaluating PA’s effects on cognitive performance reported outcome measures related to the domains of executive functioning (*n* = 10), namely, cognitive flexibility (shifting), working memory, inhibition, updating, and behavioural and emotional control. However, some studies assessed selective attention (*n* = 2), intelligence (*n* = 2), and speed of information processing (*n* = 1). 

### 3.3. Physical Activities Effect on Academic Performance

Results varied across the academic performance related studies (*n* = 9); two reported positive, significant effects, two reported insignificant effects, and five reported mixed conclusions. A brief description of intervention designs associated with the aforementioned effects is reported below. Furthermore, detailed descriptions of included studies, their characteristics, methodological processes, and key findings are summarised in Table S1. A study conducted by Gall et al. [40] increased PA through a multifaceted PA intervention design, incorporating PE lessons, active breaks, and active academic lessons. The intervention had a significant (*p* = 0.032), positive effect on academic performance compared to children receiving PE lessons in isolation. Likewise, a study conducted by García-Hermoso et al. [50] reported that increased moderate to vigorous PA has a significant (*p* < 0.001), positive effect on academic performance. Ardoy et al. [48] compared academic performance across three intervention conditions: a moderate-intensity group (moderate-intensity PE lesson for 55 min, four times weekly), a high-intensity group (high-intensity PE lesson for 55 min, four times weekly), and a control group (regular PE lessons for 55 min, twice weekly). Interestingly, the high-intensity group had a significant (*p* < 0.001), positive effect on academic performance compared to the control condition. However, no significant (*p* > 0.05) difference was observed between the moderate-intensity and control groups. Similarly, Mavilidi et al. [47] and De Bruijn et al. [49] compared the effects of different types of increased PA on academic performance and reported mixed conclusions. Mavilidi et al. [47] reported a significant (*p* = 0.045), positive relationship between active breaks and academic performance, but reported an insignificant (*p* > 0.05) relationship between cognitively engaging, active breaks and PA. In contrast, De Bruijn et al. [49] reported that both increased cognitively engaging, moderate to vigorous PE and increased moderate to vigorous PE had no significant (*p* > 0.05) overall effect on academic performance (spelling, mathematics, and reading) compared to the control condition. However, the study reported a significant (*p* ≤ 0.05) dose–response relationship between increased moderate to vigorous PA and mathematics score, and a significant (*p* ≤ 0.05) dose–response relationship between moderate to vigorous, cognitively engaging PA and mathematics and spelling performance.

Mullender-Wijnsma et al. [45] and Resaland et al. [46] also reported mixed results. The study conducted by Mullender-Wijnsma et al. [45] found no significant difference between the overall academic performance of children receiving active academic lessons and those receiving regular academic lessons (*p* > 0.05). However, a significant interaction between grade and reading and mathematics performance was reported. Interestingly, the third-grade (8–9 years) children receiving active academic lessons scored significantly (*p* < 0.01) higher than those receiving regular academic lessons for mathematics and reading performance. In contrast, the active academic lessons had no significant (*p* > 0.05) effect on second-grade (7–8 years) students’ reading scores and a significant (*p* < 0.01), negative effect on mathematics scores compared with those receiving regular academic lessons. Similarly, Resaland et al. [46] reported no significant (*p* > 0.358) intervention effect on overall academic performance but a significant (*p* = 0.005) effect between subgroup and numeracy score. Notably, the intervention group with the lowest baseline numeracy score significantly improved its post-test numeracy score compared to the control condition. The study included a multifaceted intervention design (active breaks, active academic lessons, and active homework) incorporating 165 min per week of additional PA.

Bugge et al. [55] compared the academic performance of a group receiving PE 6 days per week (4.5 h per week) and a group receiving PE 2 days per week (1.5 h). The study found no significant (*p* > 0.5) difference between academic performance of both groups following the intervention. Likewise, a study conducted by Donnelly et al. [43] compared academic performance across a group receiving a moderate to vigorous PA intervention and a group that followed the regular curriculum and found no significant (*p* > 0.5) intervention effects on mathematics, reading, and spelling performance. However, improvements were reported for both groups across all three academic performance indicators.

### 3.4. Physical Activities Effect on Cognitive Performance

Of the 14 studies that examined the relationship between PA and domains of cognitive performance: six reported positive, significant effects, while four reported insignificant effects, and four reported mixed conclusions. Succinct descriptions of intervention designs associated with these effects are provided below. Moreover, detailed descriptions of the studies, their characteristics, methodological processes, and key findings are provided in Table S1.

Studies conducted by Ronzi and Greco [52] and Phung and Goldberg, [53] found that increased participation in PA through combat sports (karate and mixed martial arts) significantly (*p* ≤ 0.05) improved executive functioning test scores when compared to the control condition. Similarly, Pan et al. [54] investigated the effects of increased PA through the medium of a specific sport (table tennis) and reported a significant (*p* < 0.01), positive intervention effect on executive functioning compared to the control condition. Kvalø et al. [44] found that children who participated in 460 min of PA per week performed significantly (*p* = 0.001) better on an executive functioning test than a group participating in 135 min of PA per week. Notably, 460 min of PA per week in Kvalø et al. [44] study was achieved through a multifaceted intervention design, incorporating active academic lessons, active breaks, PE, and active homework.

Ziereis and Jansen, [5] compared executive functioning across three conditions: a general sports group (60 min of PA, 1 day per week for 12 weeks, with an emphasis on nonspecific sporting movements), a specific sports group (60 min of PA, 1 day per week for 12 weeks, with an emphasis on specific sports), and a control group (a group that did not receive a PA intervention). Both interventions had a significant (*p* ≤ 0.05), positive effect on executive functioning compared to the control condition. However, no significant (*p* > 0.5) difference between the general and specific sports groups was reported following the 12 weeks. Moreover, Reed et al. [41] found that children participating in 45 min of generalised sport (multiactivity sport or fundamental movement skill development) five times per week performed significantly (*p* ≤ 0.05) better in eight out of 26 cognitive performance indictors than a group receiving regular PE for 30 to 50 min, once weekly.

Ardoy et al. [48] found that increased levels of moderate-intensity PE per week had no significant (all *p* ≥ 0.2) effect on cognitive performance compared to the control condition. However, increased levels of high-intensity PE per week had a positive, significant (*p* ≤ 0.001) effect on cognitive performance compared to the control condition. Congruently, Schmidt et al. [51] also compared cognitive performance (executive functioning) across three conditions: a high-intensity, cognitively engaged group (45 min of high-intensity PA, 2 days per week for 6 weeks, with an emphasis on team games with a high degree of cognitive engagement), high-intensity group (45 min of high-intensity PA, 2 days per week for 6 weeks, with an emphasis on aerobic exercise with a low degree of cognitive engagement), and a control group (45 min of low-intensity PA, 2 days per week for 6 weeks) and reported mixed results. Interestingly, cognitive flexibility (shifting) significantly (*p* ≤ 0.05) improved in the high-intensity, cognitively engaged group compared with the high-intensity and control groups. However, there was no significant (*p* > 0.5) difference between the updating and inhibition performance across the three groups. 

Pan et al. [56] compared cognitive performance (executive functioning) across two conditions: an intervention group (70 min of PA, twice per week for 12 weeks; see Table S1 for session design) and a control group (a group that did not receive any form of PA intervention). The study reported mixed results; an insignificant (*p* > 0.5) main effect of group and time on all indices of the executive functioning test was reported. However, the intervention had a significant (*p* < 0.01), positive effect on three indices of the executive functioning test compared to the control condition, namely, total correct, conceptual level response, and preservative response. Similarly, Pritchard et al. [57] reported an insignificant (*p* = 0.173) intervention effect on the first component of an executive functioning test but a significant (*p* = 0.014), positive effect on the second component of the test compared to the control condition. Notably, the intervention group received 90 min of PA (see session design in Table S1), 2 days per week for 8 weeks, whilst the control condition did not receive any form of PA intervention during the study.

Mavilidi et al. [47] and de Greeff et al. [42] investigated the effects of active breaks on cognitive performance. Mavilidi et al. [47] reported that active breaks had no significant (*p* > 0.5) effect on executive functioning compared to the control condition, whilst de Greeff et al. [42] found no significant (*p* > 0.5) intervention effect on shifting performance. Likewise, studies conducted by Gall et al. [40] and García-Hermoso et al. [50] found no significant (*p* > 0.5) intervention effect on cognitive performance compared to a control condition (group receiving regular PE lessons). Notably, Gall et al.’s [40] intervention included 180 min of additional PA, whilst the García-Hermoso et al.’s [50] study included 150 min of additional PA per week.

## 4. Discussion

This article aimed to examine the literature surrounding PA’s effect on academic performance in school children. To the author’s knowledge, this is the first study to assess PA’s effect on academic performance to include a heterogeneous participant group that is truly representative of the school community. Moreover, the authors aimed to investigate the causal factors related to the associated effects. Following the research, it can be said with confidence that a key finding in this study is that PA does not diminish academic performance in school children and potentially enhances it. Altogether, the results across the studies varied considerably. However, studies predominantly reported that PA is either positively associated with academic performance, or that there is no significant relationship between the two variables in either direction. Interestingly, Mullender-Wijnsma et al. [45] was the only article to report a negative, significant association between PA and academic performance, whilst the other articles either reported positive, significant associations (50% reported positive, significant associations) or insignificant associations (47% reported insignificant). Nevertheless, the results reported reflect those of Marques et al. [14], Ericsson and Karlsson [15], Aadland et al. [25] and Rasberry et al. [28].

An important finding to emerge from the synthesis was that various frequencies of PA were associated with improved academic performance. For example, 4 days of PA a week improved academic performance in the De Bruijn et al. [49] study, as did 5 days a week in the Reed et al. [41] study. These results are consistent with that of Rasberry et al. [28], who also reported a positive or insignificant effect of PA on academic performance irrespective of the frequency implemented. A possible explanation for this might be that the total amount of PA is more important than the accrual pattern [58]. Thus, if participants accumulated a total amount of PA that is adequate to elicit improved academic performance, how they accumulated this may not matter. However, a downfall of the De Bruijn et al. [49] and Reed et al. [41] studies is that effect size was not reported; thus, several questions surrounding this remain. Notably, future research should note the effect size so that the dose–response relationship between frequency of PA and academic performance is better understood. Moreover, further research should be undertaken to investigate if the pattern of accrual has a mediating effect on the relationship between the total amount of PA and academic performance. 

The duration of PA implemented across individual studies differentiated profusely, ranging from 15 min of PA per week to 325 min of PA per week. However, of the studies that reported positive intervention effects between PA and academic performance, most implemented PA that lasted 30–60 min. These results are significant as they collaborate with an array of literature that suggests children and adolescents should receive at least 60 min of PA per day to improve health outcomes [2,59,60,61], suggesting that schools can facilitate a child’s physical, mental, and cognitive health without concern that such endeavours deter academic performance. However, it is imperative to note that several of the included studies derived their conclusions on the basis of subjective rather than objective measures of PA. This has significant implications for the validity and reliability of their results, as subjective measures of PA are less accurate and reliable than objective measures [62]. Therefore, it is recommended that future research utilises an objective measure of PA, such as an accelerometer, so that conclusions surrounding PA’s effect on academic performance are informed by accurate data that is representative of the PA levels that took place. 

Regarding the total amount of PA, a significant finding across several studies was that increased volume of PA (total amount of PA accumulated over 1 week, frequency × duration) either did not affect academic performance or positively affected academic performance. Notably, of the studies that reported positive associations between PA and academic performance, the majority reported positive effects when the volume of PA was ≥90 per week. These findings further support the idea that the total amount of PA is a more critical factor than the frequency of PA for improved academic performance. Since most positive associations occurred when the volume of PA was ≥90 per week, irrespective of how individual studies accrued this.

No clear patterns were observed regarding the most favourable volume of PA to improve academic or cognitive performance. Therefore, on the basis of the findings in this study, the authors recommend that children receive at least 90 min of PA per week, but most desirably, their recommended levels of 60 min of PA per day, as this does not detract from academic performance and is associated with a vast amount of positive health effects [63]. Nevertheless, future research should be carried out to establish an optimal volume of PA for the improvement of academic performance. Further research could also explore the implementation of varying PA interventions and assess the differentiating effects this can have on a participants individualised academic needs.

Moreover, it is recognised that many studies manipulate independent variables such as intensity, volume, and type of PA cohesively when implementing a PA intervention design. One of the issues that emerge from this is that the actual effects of each independent variable are difficult to establish. Thus, future research should investigate the effects of volume, intensity, and type of PA in isolation so that the actual effects of each independent variable on academic performance are better understood.

It is also interesting to note that the intensity of PA had a significant effect on academic performance outcomes. Notably, across all the studies that reported moderate- to vigorous-intensity PA, 57.1% reported a positive intervention effect between moderate to vigorous PA and at least one academic performance indicator. Among the studies that reported on high-intensity PA, 100% reported a positive intervention effect between high-intensity PA and at least one academic performance indicator. These results suggest that high-intensity PA is most effective in eliciting improved academic performance. These results are significant for the implementation of increased PA in schools as it can be assumed shorter bouts of high intensity PA will be more feasible to implement alongside classroom activities than the more traditional, longer-duration, low-intensity PA interventions. These results are consistent with those attained by Mekari et al. [64], who found that high-intensity PA had a significant, positive effect on cognitive performance compared to moderate-intensity PA. A possible explanation for this might be that PA at a higher intensity causes more substantial and more enduring neurobiological changes that consequently lead to more significant improvements in academic performance [65]. Moreover, high-intensity PA was recognised in a study by Hannan et al. [66] to be more effective than moderate-intensity PA for improving aerobic fitness and cardiovascular health. This is significant as several studies state that aerobic fitness is a predictor of academic performance [67,68,69]. Therefore, it can be inferred that the high-intensity PA implemented by Ardoy et al. [48] and Schmidt et al. [51] improved aerobic fitness and, as a result, academic performance Improved congruently. Moreover, it can, thus, be suggested that specific PA may not be the single most crucial factor for improving academic performance but rather the physiological changes PA elicits. Therefore, the lens through which we view PA must be much broader. Nevertheless, it is recognised that the validity of these conclusions is questionable as they are derivative of only two studies. Thus, more research in this area is required to increase the statistical power of these results.

Interestingly, positive associations were observed across several studies that implemented a variety of different types of PA, such as karate, table tennis, mixed martial arts, team games, and cognitively engaging PA. These results are significant as they further reiterate the idea that the specific PA may not be the single most crucial factor in improving academic performance, but rather the physiological changes the PA elicits. Therefore, if the appropriate physiological changes happen, it does not matter what type of PA induces the response, as long as the response takes place. For example, aerobic fitness has been associated with academic performance outcomes [70]. Therefore, any type of PA that improves aerobic fitness may have a positive effect. Moreover, an apparent similarity across many of the efficacious types of PA was that they require a high degree of agility. For example, Pal et al. [71] stated that karate requires maximum levels of agility. Likewise, table tennis is associated with agility [72], as are many team games [73] and mixed martial arts [74]. Accordingly, it can be assumed that levels of agility may have a significant effect on academic performance. Nevertheless, future research is recommended to explore this relationship further. Regarding the optimal type of PA to improve academic performance, the authors recommend implementing various types of PA that improve agility and aerobic fitness. This is based on evidence supporting the positive relationship between these types of PA and academic performance.

### 4.1. Limitations

This present study systematically searched, reviewed, and collated the literature surrounding PA’s effect on academic performance and provided a clear and comprehensive overview of the available evidence. Moreover, 10 years of research were covered, specific eligibility criteria were followed, and a broad range of studies were considered. Nevertheless, as with most studies, the design of this current review is subject to limitations that could be addressed in future research. Most notably, the effect size was ill-reported throughout the available literature. Thus, few conclusions were made surrounding the strength of the relationship between the independent and dependent variables. Nevertheless, to the authors’ knowledge, this current review is one of the first studies to examine the relationship between FIIT of PA and academic performance. Despite effect size being ambiguous, this review enhances our understanding of the causal factors related to the associated effects and offered many areas for future research to explore; this insight into intervention design considerations is, therefore, a strength of the study. Study designs, outcome measures, and PA varied considerably across individual studies. Thus, these inconsistencies limited the number of objective conclusions and comparisons that could be made. Nevertheless, the scope of this review was deliberately broad to ensure all relevant literature was considered. Moreover, measures of academic performance differentiate profusely from country to country [37,38,39]. Therefore, we included a broad scope of academic measures to eradicate bias towards a particular country or place. Outcome measures related to academic and cognitive performance are described throughout this current review under the encompassing term “academic performance”. A possible limitation to doing this is that the intricacies of effects are not fully explored. For example, cognitive performance is indirectly associated with academic performance, whilst reading score is directly associated with academic performance; however, describing the results of these outcome measures under the encompassing term “academic performance” does not allude to this. Nevertheless, due to the range of outcome measures implemented by the individual studies, the authors decided to utilise an encompassing term such as “academic performance” to ensure the reader can easily interpret the results.

### 4.2. Practical Implications

Participant heterogeneity is one of the significant advantages of this current review. Specifically, the inclusion of differentiated participant groups ensured that this review is relevant and applicable to the school community, in which many individuals are characteristically diverse. If future research is performed, then it would be advantageous to assess a subgroup analysis to look at the academic effects of PA in specific subgroups. Nevertheless, this present study lays the groundwork for future research in this area. The findings of this research will be shared with our local and regional physical activity and public health networks so that learning from this study can benefit practitioners and policymakers as part of their programme delivery and planning. Moreover, because PA has been shown to improve and not deter academic performance, it can only be thought that such findings will influence educational reform, allowing teachers and educational leaders to integrate increased amounts of PA into the school day and national curriculum subjects. Consequently, pertinent issues such as childhood physical inactivity and childhood obesity can be addressed in schools without concern that such practices deter children’s intellectual development. This research also sheds light on optimal PA protocols for improved academic performance; accordingly, it can be utilised by teachers, policymakers, and parents as a foundational set of guidelines or benchmark of best practices to strive towards when developing their PA interventions.

## 5. Conclusions

To the authors’ knowledge, this is the first study to review the literature surrounding PA’s effect on academic performance and provide a contextualisation of the causal factors related to the associated effects. This study established that PA is either positively associated with academic performance, or that there is no significant relationship between the two variables in either direction. This study could not establish an optimal frequency of PA to improve academic performance; however, it was observed that total volume, duration, intensity, and type of PA may have a significant effect. Notably, PA levels of 90 min or more per week were associated with improved academic performance, as was PA performed at moderate to vigorous and high intensity. The optimal duration of PA was found to be 30–60 min per session, whilst a variety of efficacious sports made determining an optimal type of PA less clear, although evidence indicated that aerobic and agility-based sports were most favourable with several academic performance indicators. Moreover, several studies reported that increased time allocated to PA did not have a deleterious effect on academic performance. In fact, quite contrarily, many studies reported that increased time allocated to PA was positively associated with academic performance. We aim for this study’s findings to help inform evidence-based interventions and policies surrounding the implementation of PA in schools, whereby health promotion and optimal academic performance should be a priority. We will disseminate the outcomes of this study through our local, regional and national PA and public health networks. Nevertheless, given that investigation into the causal factors of the associated effects is in its infancy, further research is recommended to address the limitations of this current study and explore the gaps that were identified throughout. Most notably, the authors recommend investigations into the relationship between PA and academic performance by subgroup (e.g., disabled, gender, and race), an examination of the relationship between agility and academic performance, and further research that explores the effects of the FITT of PA on academic performance outcomes.

## Figures and Tables

**Table 1:** Databases searched and search terms employed.

Databases Searched	Academic Performance-Related Search Terms Employed	Physical Activity-Related Search Terms Employed	Population-Related Search Terms Employed
Pubmed	Academic performance	Physical activity	Children
SportDiscus	Cognitive functioning	Exercise	School-aged children
Science direct	Academic achievement	Play	Key stage 1
	Standardised test scores	Physical activity intervention	Key stage 2
	Student engagement	Exercise intervention	Key stage 3
	Cognitive performance	Sport	Child
	Student achievement	PE	Primary school children
	Mathematics performance	Sport participation	Adolescents
	Reading performance	Active breaks	Young children

**Table 2:** Academic performance-related keywords.

Academic Performance-Related Keywords
Academic performance
Reading score/performance/ability
Mathematics score/performance/ability
National test
Intelligence test scores
Cognitive functioning *
Executive functioning *
Academic grades
Grades
Student achievement
Standardised national test
Cognitive outcomes *

* Cognitive and executive functioning are broad terms encompassing numerous mental processes involved in multiple cognitive tasks. Therefore, if appropriate, any keywords relating to these domains were also included when documenting outcome measures and findings (e.g., working memory, on-task behaviour, decision making, and selective attention).

## Data Availability

The data presented in this study are available in the article.

## Supplementary Materials

The following supporting information can be downloaded at https://www.mdpi.com/article/10.3390/children10061019/s1; Table S1. Academic/cognitive performance and physical activity, and article descriptive results.
