# Supplementary material for: The Effects of Physical Activity on Academic Performance in School-Aged Children: A Systematic Review
Source: Children (Basel). 2023 Jun 5;10(6):1019. doi: 10.3390/children10061019 (PMC10297707; doi:10.3390/children10061019)
Supplement: Supplementary file 1 [file children-10-01019-s001.zip › children-2253835-supplementary.pdf]

TABLE S1. ACADEMIC/COGNITIVE PERFORMANCE AND PHYSICAL ACTIVITY, AND ARTICLE DESCRIPTIVE RESULTS.

AUTHOR	Study design	Country of origin	Study Sample	Study details	Dependant variable of Interest (relating to academic/cognitive performance)	Key findings (relating to academic/cognitive performance and PA)
ARDOY, ET AL., [48]	A Group-Randomised Controlled Trial<br><br>(School-based)	Spain	67 participants<br><br>43 boys, 24 girls<br><br>Mean age –<br><br>N/A<br>12-14 years old<br><br>(Non-disabled)	*Independent variable of Interest (relating to PA) -*<br>School-based PA, PE with increased intensity and/or volume.<br><br>*Intervention characteristics –*<br>G1 – F – 4 days per week // 4 months<br>I – moderate intensity<br>T – 55 mins per session<br>T- moderate-intensity PE<br><br>G2 – F - 4 days per week // 4 months<br>I – high intensity<br>T – 55 minutes per session<br>T – high-intensity PE<br><br>CG – F – 2 days per week // 4 months<br>I – N /A<br>T – 55 minutes per session<br>T – Regular PE, which conforms to the national laws on PE.	*Encompassing dependant variable of Interest –*<br>Cognitive performance, academic performance<br>*Specific outcome measure(s)-*<br>Cognitive performance - the medium version of the Spanish Overall and Factorial Intelligence Test (IGF-M).<br>Academic performance – Grades in core academic subjects (e.g., mathematics and language) and other subjects (e.g. social sciences and music)	*The principal outcome –* An increased number of high-intensity PE sessions per week has an overall significant, positive effect on academic and cognitive performance.<br>On the contrary, an increased number of moderate-intensity PE sessions per week had no significant effect on academic and cognitive performance.<br>*Notable findings / supporting evidence*<br>G2 significantly improved cognitive performance when compared to CG and G1 (all $p \leq 0.001$, and verbal reasoning $p = 0.02$)<br>No significant effects were observed between G1 and CG for cognitive performance indicators (all $p \geq 0.2$).

						G2 had a significant, positive effect on academic performance (across all subjects) when compared to CG and G1 ($p < 0.001$).
BUGGE, ET AL., [55]	A quasi-experimental research design<br><br>(School-based)	Denmark	1181 participants<br><br>552 boys, 629 girls<br><br>Mean age – 8.39 (SD-1.44)<br><br>(Non-disabled)	*Independent variable of Interest (relating to PA)* -<br>School-based PA, increased volume of PE lessons<br><br>*Intervention characteristics –*<br>G1 – F – 6 days per week (or a minimum of 4.5 hours total per week) // 2- to 6- years<br>I – N/A<br>T- 45 minutes per session<br>T – regular PE lessons<br><br>CG - F - 2 days per week (or a total of 1.5 hours per week) // 2- to 6- years<br>I – N / A<br>T – 45 minutes per session<br>T- Regular PE Lessons	*Encompassing dependant variable of Interest –*<br>Academic performance<br>*Specific outcome measure(s)-*<br>- Standardised national test results – Danish national test system	*The principal outcome* – No significant differences were found between the two conditions (G1 and CG) in academic performance.<br>*Notable findings / supporting evidence –*<br>G1 had no significant effect on academic performance compared to CG ($p > 0.5$)
DE BRUIJN, ET AL., [49]	A cluster-randomised control trial<br><br>(School-based)	Netherlands	891 participants<br><br>420 boys, 471 girls<br><br>Mean age – 9.17 (SD-0.66)	*Independent variable of Interest (relating to PA)* -<br>School-based PA, Increased volume of PE and//or increased volume of	*Encompassing dependant variable of Interest –*<br>Academic performance<br>*Specific outcome measure(s)-*<br>Standardised test battery results (used in the Netherlands).	*The principal outcome* – Overall, there was no significant difference between the intervention and control group's

			(Non-disabled)	cognitively engaging PE.<br><br>*Intervention characteristics –*<br>G1- f – 4 days per week // 14 weeks<br>I – moderate to vigorous<br>T – 30 minutes per session<br>T – moderate to vigorous PE with a low degree of cognitive engagement<br><br>G2 – F – 4 days per week // 14 weeks<br>I – moderate to vigorous<br>T – 30 minutes per session<br>T – moderate to vigorous PE with a high degree of cognitive engagement<br><br>CG – F – 2 days per week // 14 weeks<br>I – n/a<br>Time – N/A<br>Type – regular PE in accordance with their national curriculum.	Mathematics, spelling and reading test results were also assessed	academic performance. However, a dose-response relationship was recognised between moderate to vigorous PA and academic performance. Notably, post-test results revealed that increased volume of moderate to vigorous activity (G1) was associated with better mathematics scores. Whilst increased volume of moderate to vigorous, cognitively engaging PA (G2) is associated with better mathematics and spelling performance.<br>*Notable findings / supporting evidence –*<br>A comparison between G1 and CG revealed that there was no significant difference between the conditions post-test reading performance (p= .60), mathematics performance (p= .58) and spelling performance (p= .77). Likewise, a comparison

						between G2 and CG found no significant difference between the group's post-test reading performance (p= .19), mathematics performance (p= .88) and spelling performance (p= .13). Increased volume of moderate to vigorous PA (both with and without cognitive engagement) was positively associated with post-test mathematics performance (p≤0.05), Increased volume of moderate to vigorous, cognitively engaging PA was positively associated with spelling performance (p≤0.05),
DE GREEFF, ET AL., [42]	A randomised controlled trial<br><br>(School-based)	Netherlands	499 participants<br><br>226 boys, 273 girls<br><br>Mean age – 8.1 (SD-0.7)<br><br>(Non-disabled)	*Independent variable of Interest (relating to PA)* – Classroom-based PA, physically active academic lessons<br><br>*Intervention characteristics* – G1 – F – 2 days per week // 2 years	*Encompassing dependant variable of Interest* – Cognitive performance (Executive functioning)<br><br>*Specific outcome measure(s)*- Inhibition – Golden Stroop test<br>Working memory – Digit span backward and	*The principal outcome* –No significant differences were observed for both conditions' executive function test scores. Similarly, no significant differences were identified between both groups' executive

				I – Moderate to vigorous<br>T – 30 minutes<br>T – physically active academic lessons whereby maths and language development were taught alongside PA. For example, specific exercises were performed for every academic task (e.g., a star jump for each letter spelt in the word "cat". This was incorporated as an addition to regular curriculum activities<br><br>CG – received their regular curriculum activities and lessons.	Visual Span backward tests.<br>Cognitive flexibility –<br>a modified version of the Wisconsin card sorting test (M-WCST)	functioning test scores at each time point (following year one and following year two).<br>*Notable findings / supporting evidence –*<br>The study indicates that a positive but insignificant effect was observed for both groups' executive functioning test scores (all p > 0.05). Therefore, the study did not provide the p values for both groups' improvements.<br>Three models were used to examine the intervention's effects on executive functioning. (A covariate model, model 1 and model 2)<br>Each model exemplified no significant difference between the control and intervention group's executive functioning test scores. For example, model 2 shows there was no significant difference between both groups' Golden Stroop test scores (p=0.250), Digit

						Span backward test score (p= 0.732), Visual backward test score (p=0.672) and M-WCST score (p=0.937).
DONNELLY, ET AL., [43]	A cluster-randomised controlled trial<br><br>(School-based)	United States of America	584 participants<br><br>284 boys, 300 girls<br><br>Mean age – 8.1 (SD-0.6)<br><br>(Non-disabled)	*Independent variable of Interest (relating to PA)* – Mixed design (integrating both school-based and class-based methods), physically active academic lessons, and increased volume of PE.<br><br>*Intervention characteristics* – G1 – F – 5 days per week // 3 years<br>I – moderate to vigorous intensity<br>T – Component 1 – 60 minutes // one day per week<br>Component 2 – 20 minutes per day // five days per week<br>T – Component 1 – PE lesson<br>Component 2 – physically active academic lesson/instruction (10 minutes in the morning, 10 minutes in the afternoon) // five days per week	*Encompassing dependant variable of Interest* – Academic performance<br>*Specific outcome measure(s)*- Wechsler Individual Achievement Test-third edition (WIAT-III) (Improvements or detriments to the participant's results).	*The principal outcome* – G1 neither improved nor decreased academic performance over the three years.<br>Academic performance improved for both groups; however, there was no significant difference between group effects.<br>Notably, G1's target of an additional 100 min/per week of PA was not achieved. Instead, the group achieved an average of 55 mins/per week.<br>*Notable findings / supporting evidence* – Academic performance improved for both groups, although there were no significant differences between-group differences in mathematics (p=0.082), reading (p=0.056) and spelling (p=0.366) performance

				Control group – did not receive any form of PA intervention		following the three years. Likewise, a linear mixed model analysis revealed that when accounting for baseline differences in academic performance, ethnicity, cardiovascular fitness and income, G1 did not significantly affect academic performance.
GALL, ET AL., [40]	A cluster-randomised controlled trial<br><br>(School-based)	South Africa	663 participants<br><br>324 boys, 339 girls<br><br>Mean age – 9.25 (SD-1.00)<br><br>(Non-disabled)<br><br>Participant characteristics - All eligible participants were from disadvantaged neighbourhoods	*Independent variable of Interest (relating to PA)* – Mixed design (integrating both school-based and classroom-based methods), increased volume of PA<br><br>*Intervention characteristics* – G1- f – 3-5 days per week // 10 weeks<br>I – N/A<br>T –There are multiple components to the intervention design; however, the study does not document the time allocated for each respective component.	*Encompassing dependant variable of Interest* – Academic performance, cognitive performance (cognitive functioning).<br>*Specific outcome measure(s)-* Academic performance – Academic outcomes were conceptualised using a routine EoYR. This is an average score taken from four subjects, home language (Xhosa or Afrikaans); first additional language (English); mathematics; and life skills.<br>Cognitive functioning – Selective attention - The d2 test, developed by Brickenkamp et al.	*The principal outcome* – The intervention had a significant, positive effect on academic performance. However, no significant effect for selective attention was observed.<br>*Notable findings / supporting evidence* – When protentional cofounders and baseline results have been accounted for, G1 has a positive, significant effect on academic performance (p=0.032), although no effect was found for selective attention (concentration performance;

				Component 1 – 45 minutes // 2 days per week<br>Component 2 – 45 minutes // 2 days per week<br>Component 3 – N/A<br>Component 4 – N/A<br>T – Component 1 – PE lessons<br>Component 2 – move to the music lesson<br>Component 3 – active breaks<br><br>CG – received regular PE; however, they did not receive any form of PA intervention.		p= 0.469; error percentage; p= 0.237).<br>Notably, this was due to G1's academic performance remaining stable whilst the CG's academic performance decreased over the intervention period.
García-Hermoso, et al., [50]	A randomised controlled trial<br><br>(School-based)	Chile	170 participants<br><br>96 boys, 74 girls<br><br>Mean age –<br><br>N/a<br><br>8-10 years old<br><br>(Non-disabled)	*Independent variable of Interest (relating to PA)*<br>Extracurricular PA (before school intervention), increased volume of PA.<br><br>*Intervention characteristics –*<br>G1- F – 5 days per week // 8 weeks<br>I – Moderate to vigorous intensity<br>T – 30 minutes<br>T – A before school game-based PA intervention (8-8.30 am)<br><br>Notably, the intervention was implemented alongside the	*Encompassing dependant variable of interest –*<br>Academic performance, cognitive performance (cognitive functioning)<br><br>*Specific outcome measure(s)-*<br>Academic performance – Mathematics and English language grades<br>Cognitive functioning – Attention capacity - d2 Test of Attention (d2)	*The principal outcome –* The intervention significantly improved academic performance compared to the control condition. However, the intervention had no significant effect on cognitive functioning.<br>*Notable findings / supporting evidence –*<br>No significant differences were found for concentration (p=0.535) and selective attention (p=0.125) performance<br>Once adjusted for covariates, G1 had a

				participant's regular PE lessons (120 minutes // per week)<br><br>CG – received their regular pe lessons of 120 minutes per week		significant, positive effect on language and mathematics performance (all (p<0.001)
GRECO & DE RONZI, [52]	A randomised controlled trial<br><br>(non-school-based)	Italy	28 participants<br><br>24 boys, 4 girls<br><br>Mean age- 9.25 (SD- 1.00)<br><br>(Disability group)<br><br>Participant characteristics- All eligible participants were formally diagnosed with autism spectrum disorder (ASD).	*Independent variable of Interest (relating to PA)* Extracurricular PA, Increased volume of PA. Specifically, karate training.<br><br>*Intervention characteristics –*<br><br>G1 – F – 2 days per week // 12 weeks<br>I – N / A<br>T – 45 minutes per session<br>T – Karate training that consisted of numerous cognitive and physical components<br><br>CG – did not receive any intervention and maintained their regular routines	*Encompassing dependant variable of Interest –* Cognitive performance (executive functioning)<br>*Specific outcome measure(s)-* Behaviour Rating Inventory of Executive Function (BRIEF) Notably, this is an 86-item parent and teacher rating scale formulated to assess executive functioning and self-regulation of children and adolescents (5-18 years of age). The BRIEF utilises multiple clinical scales to encompass many domains of executive functioning, these being; the Behaviour Regulation Index (BRI;<br>scales: inhibit, self-monitor), Emotion Regulation Index	*The principal outcome –* The intervention had a significant, positive effect on the participant's executive functioning. In contrast, no significant differences were found across all outcome variables for the control condition.<br>*Notable findings / supporting evidence –* Post-hoc tests revealed that G1 had a significant, positive effect on BRI (p<0.001), ERI (p<0.001) and CRI (p<0.001). Consequently, G1 significantly improved the participant's Global Executive Composite score (p=0.003).

					(ERI; scales: shift, emotional control), and Cognitive Regulation Index (CRI; scales: initiate, working memory, plan/organize, task-monitor, organization of materials). The three indexes were then combined to make a Global Executive Composite score. Participant Scores from each scale were curated, and a mean result was taken for each participant. Notably, a high score indicates poorer executive functioning.	
KVALØ, ET AL., [44]	A Group-Randomized Controlled Trial<br><br>(School-based)	Norway	449 participants<br><br>230 boys, 219 girls<br><br>Mean age – N/A<br><br>9-10 years old<br><br>(Non-disabled)	*Independent variable of Interest (relating to PA)* - Mixed design (integrating school-based, class-based extracurricular methods): increased volume of PA (combined through a variety of PA interventions).<br><br>*Intervention characteristics* G1 – F – due to multiple components to the intervention design, total time per week has been noted	*Encompassing dependant variable of Interest* – Cognitive performance (executive functioning)<br>*Specific outcome measure(s)*- Selective attention, response inhibition, self-control and mental speed were assessed via the Stroop Golden colour-word test. Initiation, efficient organisation of verbal retrieval and recall, and self-monitoring were measured Verbal semantic fluency test.	*The principal outcome* – The intervention group had a significant, positive effect on executive functioning compared to the control condition.<br>*Notable findings / supporting evidence* –There was a significant, positive main effect across G1's (time) for executive function (p=0.001*)* however, there was a non-significant group x time interaction (p=0.057)

				– 325 minutes per week // 10 months<br>I – n/a<br>T – Component 1 – 45 minutes // 2 days per week<br>Component 2 – 10 minutes // 5 days per week<br>Component 3 – 10 minutes // 5 days per week<br>T – Component 1 – physically active academic lessons<br>Component 2 – physically active breaks<br>Component 3 – physically active homework<br><br>Notably, all the above components were integrated alongside regular 135 mins/ per week of pe.<br><br>CG – received regular 135 mins//per week of curriculum-based pe	Attention, psychomotor execution speed, and mental flexibility were assessed via Trail making (TMT).<br>working memory was measured using the Forward and backward digit span test, which derived from the intelligence test WAIS-IV<br>Notably, Test results from all the tests were then standardised and united to provide a composite executive functioning test score for each participating child pre-post intervention.	
MAVILIDI, ET AL.,[47]	Group randomised controlled trial<br><br>(School-based)	Australia	87 participants<br><br>53 boys, 34 girls<br><br>Mean age - 9.11 (SD-.62)	*Independent variable of Interest (relating to PA)* -<br>Class-based physically active breaks and cognitively engaging	*Encompassing dependant variable of Interest* – Cognitive performance (executive functioning), academic performance<br>*Specific outcome measure(s)-*	*The principal outcome* – Active breaks had a positive, significant effect on academic performance, whilst active breaks with a cognitive

			(Non-disabled)	physically active breaks.<br><br>*Intervention characteristics*<br>G1 – F – 3 days per week // 4 weeks<br>I – moderate to vigorous<br>T – 5 minutes<br>T – physically active breaks conducted throughout a lesson<br><br>G2 - 3 days per week // 4 weeks<br>I – moderate to vigorous<br>T – 5 minutes<br>T – physically active breaks with a cognitive element conducted throughout a lesson.<br><br>CG – received their regular mathematics lessons without any PA/ physically active breaks.	*Academic achievement – measurement of the student's mastery of the basic facts test (Stage 2 version of the Individual Basic Facts Assessment Tool)*<br>*Executive functioning–* Inhibition - Erikson Flanker test.<br>working memory - version (2-back) of the "n-back task."	element had a positive, non-significant effect on academic performance. However, both interventions had no significant effect on executive functioning (inhibition and working memory)<br>*Notable findings / supporting evidence –* significant group by time effects were observed for mathematics performance between G1 and CG (p=0.045)<br>Non-significant group by time effects were observed for mathematics performance between G2 and CG, G1 and G2. Non-significant group by time effects were observed for participant's inhibition or working memory (all groups)
MULLENDER-WIJNSMA, ET AL.,[45]	A quasi-experimental research design that included a control condition<br><br>(School-based)	Netherlands	228 participants<br><br>122 boys, 106 girls<br><br>Mean age – 8.1 (SD-N/A)<br><br>(Non-disabled)	*Independent variable of Interest (relating to PA) -*<br>Class based PA, physically active lesson design<br><br>*Intervention design*	*Encompassing dependant variable of interest –* Academic performance<br>*Specific outcome measure(s)-* Mathematics-Tempo-Test-Rekenen (Speed Test Arithmetic).	*The principal outcome –* The intervention had a non-significant overall effect on academic performance. However, a significant interaction between grade

				G1- F – 3 days per week // 21 weeks<br>I – moderate to vigorous intensity<br>T – 20-30 minutes<br>T – physically active academic lessons whereby PA is implemented alongside academic instruction. specifically, mathematics and language development<br><br>CG – received regular academic lessons/instruction in congruence with the national curriculum.	Reading - Ee´n-Minuut-Test (1-Minute Test)	and mathematics and reading scores was observed. Interestingly, third-grade students in the intervention group scored significantly higher than the control for mathematics and reading performance. In contrast, second-grade students in the intervention group scored significantly lower than the control group for mathematics and were comparable for reading scores.<br>*Notable findings / supporting evidence –*<br>once pre-test and grade differences were controlled, no significant main effects were observed; however, there was a significant interaction between intervention and grade for mathematics ($p<0.05$) and reading score ($p<0.05$)<br>G1 had a positive, significant effect on mathematics ($p<0.01$) and

						reading (p<0.01) performance in third-grade students.<br>G1 had a negative, significant effect on mathematics performance (p<0.01) and an insignificant effect on reading performance in second-grade students (p>0.01)
PAN, ET AL., [54]	A randomised controlled trial<br><br>(non-school based)	Taiwan	32 participants<br><br>32 boys<br><br>Mean age-group 1 – 8.93 (SD-1.49)<br><br>Mean age-CG- 8.87 (SD-1.56)<br><br>(Disability group) Participant characteristics- all eligible participants were formally diagnosed with ADHD.	*Independent variable of Interest (relating to PA)* – Extracurricular PA, Increased volume of PA, specifically table tennis.<br><br>*Intervention characteristics*<br>G1 – F – 2 days per week // 12 weeks<br>I – N /A<br>T – 70 minutes per session<br>T – table tennis emphasises developing the motor skills involved in executive functioning.<br><br>CG – continued their regular routines and did not receive any form of PA intervention<br><br>Notably, a crossover	*Encompassing dependant variable of Interest* – Cognitive performance (Executive functioning)<br>*Specific outcome measure(s)-*<br>The children's version of the Stroop Color and Word Test for ages 5–14 years.<br>Notably, Measurements were taken pre-intervention, following phase 1 and post-intervention (following phase 2)	*The principal outcome* – The intervention had a significant, positive effect on executive functioning.<br>*Notable findings / supporting evidence* –<br>G1 had a significant, positive main effect on Stroop color word test score from time point 1 to time point 2 (p<0.01)<br>A significant group by time interaction was also observed (p<0.01)

				design was implemented for the purpose of the study; therefore, when the respective groups swapped, the total study time was 24 weeks. Each 12-week period was documented as either phase 1 or 2.		
PAN, ET AL., [56]	A randomised controlled trial<br><br>(non-school based)	China	22 participants<br><br>22 boys<br><br>Mean age- 9.08 (SD- 1.75)<br><br>(Disability group)<br><br>Participant characteristics- all eligible participants were formally diagnosed with ASD	*Independent variable of Interest (relating to PA)* - Extracurricular PA, increased volume of PA<br><br>*Intervention characteristics*<br>G1 – F – 2 days per week // 12 weeks<br>I – N/A<br>T – 70 minutes per session<br>T – PA activities following this format; warm-up (5 min), motor skills (20 min), motor skill training alongside components that will develop executive function (20 min), group games (20 min), and cool-down (5 min)<br><br>CG – continued their regular	*Encompassing dependant variable of Interest* – Cognitive performance (executive functioning)<br>*Specific outcome measures*<br>The computer version of the WCST test, formally titled the Wisconsin Card Sorting Test. Notably, Measurements were taken pre-intervention, following phase 1 and post-intervention (following phase 2)	*The principal outcome* – No significant main effects of group or time on all indices of WCST were observed. However, the intervention significantly improved three indices of executive functioning. Notably, interactions in the group by time differences for total correct, conceptual level response and preservative response were observed.<br>*Notable findings / supporting evidence* – significant group by time differences for total correct (p<0.01), conceptual level response (p<0.01), and preservative response

				routines and did not receive any form of additional PA intervention.<br><br>Notably, a crossover design was implemented for the purpose of the study; therefore, when the respective groups swapped, the total study time was 24 weeks. Each 12-week period was documented as either phase 1 or 2.		(p<0.01) were observed<br>Phase 1 – G1 significantly improved total correct (p<0.01), conceptual level response (p<0.01), and preservative response (p<0.01) from time point 1 to 2.<br>Phase 2 – G1 significantly improved total correct (p<0.01), and conceptual level response (p<0.01) from time points 2 to time points 3.
PHUNG & GOLDBERG, [53]	A randomised controlled trial<br><br>(non-school based)	N/A	34 participants<br><br>28 boys, 6 girls<br><br>Mean age- 9.34 (SD- 1.08)<br><br>(Disability group)<br><br>Participant characteristics- all eligible participants were formally diagnosed with ASD	*Independent variable of Interest (relating to PA)* – Extracurricular PA, increased volume of PA. Specifically, mixed martial arts (MMA)<br><br>*Intervention characteristics -*<br><br>G1- F – 2 days per week // 13 weeks<br>I – N/A<br>T – 45 minutes per session<br>T.- MMA training that emphasised developing three core executive functions: behavioural inhibition, working	*Encompassing dependant variable of Interest* – Cognitive performance (executive functioning)<br>*Specific outcome measure(s)-*<br>The hearts and flowers test behavioural inhibition, working memory, and cognitive flexibility.<br>Notably, this test used two dependent variables to analyse executive functioning: accuracy and response time (in milliseconds, Ms).<br>The Behaviour Rating Inventory of Executive Function (BRIEF-2) was also used to measure executive	*The principal outcome* – The results revealed that the intervention had a significant, positive effect on the three indices of executive functioning (behavioural inhibition, working memory and cognitive flexibility) compared to the control. This is because the results revealed that the intervention had a significant, positive effect on the accuracy findings of the congruent block (which tests working

				memory, and cognitive flexibility.<br><br>CG – continued their regular routines and did not receive any form of PA intervention.	functioning. This 86-item parent rating scale assesses executive functioning and self-regulation in children and adolescents (aged 5-18). This test assesses executive functioning across three indexes; Behaviour Regulation Index (BRI; scales: inhibit, self-monitor), Emotion Regulation Index (ERI; scales: shift, emotional control), and Cognitive Regulation Index (CRI; scales: initiate, working memory, plan/ organize, task-monitor, organization of materials) and result sets are combined to obtain a Global Executive Composite (GEC) score.	memory) and mixed block (which tests working memory, cognitive flexibility and behavioural inhibition).<br>*Notable findings / supporting evidence* –<br>G1 had a significant, positive main effect of time for accuracy in the in-congruent block (p=0.00) and mixed block (p=0.01). However, an insignificant main effect of time for accuracy in the congruent block (p=0.84) was revealed.<br>A significant interaction between the group and predicted accuracy over time was found for the mixed and congruent blocks.<br>However, no significant interaction between the group and predicted accuracy over time was found in the in-congruent block.
PRITCHARD, ET AL., [57]	A randomised controlled trial	Canada	30 participants<br><br>14 boys, 16 girls	*Independent variable of Interest (relating to PA)* –	*Encompassing dependant variable of Interest* – Cognitive performance (executive functioning)	*The principal outcome* – Mixed results: the intervention had a significant, positive effect

	(non-school based)		Mean age – 10.2 (SD- .4)<br><br>(Disability group)<br>Participant characteristics- all eligible participants were formally diagnosed with foetal alcohol syndrome	Extracurricular PA, increased volume of PA.<br><br>*Intervention characteristics* –<br>G1- F – 2 days per week // 8 weeks<br>I – N/A<br>T – 90 minutes per session<br>T – PA intervention following this sequence: warm-up, 3x15 minutes of station activities (specified on the day), 20 minutes of child or caregiver directed physical activities, cool down.<br><br>CG - continued their regular routines and did not receive any form of PA intervention.	*Specific outcome measure(s)-*<br>A children's colour trails test (CCTT). two components of the CCTT results were evaluated: CCTT1 and CCTT2	on the CCTT2 test results. However, a positive but insignificant effect on CCTT1 test results.<br>*Notable findings / supporting evidence* –<br>G1 had no significant effect on the T scores for the CCTT1 (p=0.173) from time point 1 to 2.<br>G1 had a significant, positive effect on the T scores for the CCTT2 (p=0.014) from time point 1 to time point 2.
REED, ET AL., [41]	A randomised control trial.<br><br>(School-based)	United States of America	470 participants<br><br>236 boys, 234 girls<br><br>Mean age – experimental group – 10.2 (SD-2.3)<br><br>Mean age- the control group – 11.2 (SD-1.9)<br><br>(Non-disabled)	*Independent variable of Interest (relating to PA)* –<br>School-based PA, increased volume of PE.<br><br>*Intervention characteristics* –<br>G1 – F – 5 days per week // 8 months<br>I – N/A<br>T – 45 minutes per session<br>T – PE Lessons with an emphasis on	*Encompassing dependant variable of Interest* –<br>Cognitive performance<br>*Specific outcome measure(s)-*<br>Perceptual speed test - perceptual Speed Test developed by Salthouse<br>Fluid intelligence - standard Progressive Matrices (SPM) test designed by Raven, Raven and Court	*The principal outcome* - The intervention had a significant, positive effect on 8 of the 26 cognitive measures compared to the control condition.<br>*Notable findings / supporting evidence* –<br>G1 had a significant, positive effect on elementary school males'

			Participant characteristics - predominantly African American participants	fundamental movement skills or multi-activity sports dependent upon the age-group<br><br>CG – received their regular 30/50-minute PE lesson once weekly.		section D score (p=0.02) for the SPM test compared to CG.<br>G1 had a significant, positive effect on the elementary school females' perceptual speed test score in the sec.2 test (p=0.006) and sec.3 test (p=0.005) score compared to CG.<br>G1 significantly improved the middle school female SPM score in section B (p=0.03), C (p=0.002), D (p=0.003) and E (p=0.001) compared to CG.<br>G1 had a significant, positive effect on the middle school males' SPM score in section E (p=0.002) compared to CG.
RESALAND, ET AL., [46]	A cluster-randomised control trial<br><br>(School-based)	Norway	1129 participants<br><br>588 boys, 541 girls<br><br>Mean age-10.2 (SD-0.3)<br><br>(Non-disabled)	*Independent variable of Interest (relating to PA)* - Mixed design (integrating school-based, class-based extracurricular methods); increased volume of PA<br><br>*Intervention characteristics-*	*Encompassing dependant variable of Interest* – Academic performance<br>*Specific outcome measure(s)-* Mathematics, reading and English - standardised Norwegian national test results administered by The Norwegian Directorate for	*The principal outcome* – The intervention had no significant effect on academic performance (mathematics, reading and English)<br>*Notable findings / supporting evidence* –<br>G1 had no significant effect on

				G1 – F- multiple components to the intervention design; therefore, the total time per week has been noted – 165 minutes per week extra PA per week // 7 months<br>I – N/A<br>T – component 1 – 30 minutes per session // 3 times per week<br>Component 2 – 5 minutes per day // 5 times per week<br>Component 3 – 10 minutes per day // 5 times per week<br>T – component 1 – physically active academic lesson<br>Component 2 – active break<br>Component 3 – active homework.<br>Notably, the G1 intervention was incorporated alongside the pupil's regular 135 mins of PE per week.<br><br>CG – F – 135 mins of PA per week // 7 months<br>I – N/A<br>T – N/A<br>T – received regular PE lessons in	Education and Training (NDET).	academic performance ($p > 0.358$) However, subgroup analyses found a significant improvement for those who had the lowest baseline numeracy score ($p=0.005$)

				accordance with the national curriculum.		
SCHMIDT, ET AL., [51]	A Group-Randomized Controlled Trial<br><br>(School-based)	Switzerland	181 participants<br><br>82 boys, 99 girls<br><br>Mean age - 11.35 (SD-0.60)<br><br>(Non-disabled and disability group)<br><br>Participant characteristics- the study included 8 participants formally diagnosed with ADHD.	*Independent variable of Interest (relating to PA) -* School-based, cognitively engaging PE.<br><br>*Intervention characteristics -* G1- F– 2 days per week // 6 weeks<br>I - high intensity.<br>T – 45 minutes per session<br>T – Team games with a high degree of cognitive engagement<br><br>G2 - F – 2 days per week // 6 weeks<br>I – high intensity<br>T - 45 minutes per session<br>T- aerobic exercise with a low degree of cognitive engagement<br><br>CG – F- 2 days per week // 6 weeks<br>I – low intensity<br>T - 45 minutes per session<br>T- received regular PE with a low degree of physical exertion/cognitive engagement	*Encompassing dependant variable of Interest –* Cognitive performance (executive functioning)<br>*Specific outcome measure(s)-* Updating - nonspatial n-back test<br>Shifting was assessed by scaffolding the Flanker test and increasing the number of independent variables within the test. It was therein requiring the children to adapt (shift) their thinking.	*The principal outcome –* The team games with the cognitive engagement group significantly improved executive functioning compared to the aerobic group with low cognitive engagement and the control group. Specifically, the team games with the cognitive engagement group had a significant, positive effect on shifting performance<br>.<br>*Notable findings/supporting evidence –* The three groups did not differ in terms of change in updating and inhibition performance. However, shifting performance was significantly different between groups (p=0.027). Post-hoc tests revealed the most significant

						improvement in shifting performance was in G1, than in G2 (p=0.39) and CG (p=0.012). G2 and CG did not differentiate from each other (p=5.44)
Ziereis & Jansen [5]	A randomised controlled trial<br><br>(non-school-based)	Germany	43 participants<br><br>32 boys, 11 girls<br><br>Mean age – 9.45 (SD- 1.43)<br><br>(Disability group)<br><br>Participant characteristics- all eligible participants were formally diagnosed with ADHD.	*Independent variable of Interest (relating to PA)* Extracurricular PA, increased volume of PA, Different modalities of PA.<br><br>*Intervention characteristics –*<br>G1- F – 1 day per week // 12 weeks<br>I – N/A<br>T – 60 minutes per session<br>T – PA activities with an emphasis upon non-specific sporting movements transferrable to several sporting domains (e.g., throwing, catching, jumping).<br><br>G2 – 1 day per week // 12 weeks<br>I – N/A<br>T – 60 minutes per session<br>T – PA activities with an emphasis on specific sports (e.g.,	*Encompassing dependant variable of Interest –* Cognitive performance (executive functioning)<br><br>*Specific outcome measure(s)-* Verbal working memory- The digit span (forwards/backwards) and the letter–number-sequencing task of the HAWIK – IV.<br>Visio-spatial working memory - Corsi block tapping test	*The principal outcome –* Both interventions had a significant, positive effect on executive functioning compared to the control condition.<br>*Notable findings / supporting evidence –* The results revealed a significant, positive main effect for time for; index score WM (p< 0.001), digit span forwards score (p< 0.001) and letter-number sequencing score (p< 0.05)<br>A significant, positive group x time interaction was also found for index score WM (p< 0.001), digit span backward score (p< 0.05) and letter number sequencing score (p< 0.01)

				swimming, gymnastics, climbing). CG – continued with their regular routines and did not receive any additional PA.		
